# Effects of *Dendropanax morbiferus* Leaf Extract on Sleep Parameters in Invertebrate and Vertebrate Models

**DOI:** 10.3390/antiox12101890

**Published:** 2023-10-21

**Authors:** Kayoung Ko, Yejin Ahn, Ga Yeong Cheon, Hyung Joo Suh, Yun Jae Cho, Sung-Soo Park, Ki-Bae Hong

**Affiliations:** 1 Department of Food Science and Nutrition, Jeju National University, Jeju 63243, Republic of Korea; lv007@jejunu.ac.kr (K.K.); rk2580dud@jejunu.ac.kr (G.Y.C.); 2 Research Group of Functional Food Materials, Korea Food Research Institute, Wanju-gun 55365, Republic of Korea; a.yejin@kfri.re.kr; 3 Department of Integrated Biomedical and Life Science, Graduate School, Korea University, Seoul 02841, Republic of Korea; suh1960@korea.ac.kr; 4 R&D Center, JEJUPANATEK Inc., Jeju 63243, Republic of Korea; a@jejubon.com

**Keywords:** *Dendropanax morbiferus*, antioxidants, animal models, sleep, neuromodulation

## Abstract

*Dendropanax morbiferus* is highly valued in traditional medicine and has been used to alleviate the symptoms of numerous diseases owing to its excellent antioxidant activity. This study aimed to evaluate the sleep promotion and related signaling pathways of *D*. *morbiferus* extract (DE) via behavioral analysis, molecular biological techniques, and electrophysiological measurements in invertebrate and vertebrate models. In *Drosophila*, the group treated with 4% DE experienced decreased subjective nighttime movement and sleep bout and increased total sleeping time. Moreover, substantial changes in locomotor activity, including distance moved, velocity, and movement, were confirmed in the 4% DE-treated group. Compared to *Drosophila* in which insomnia and oxidative stress were induced by exposure to 0.1% caffeine, the DE-treated group improved sleep-related parameters to the level of the normal group. In the *Drosophila* model, exposure to 4% DE upregulated the expression of gamma-aminobutyric acid (GABA)-related receptors and serotonin receptor (5-HT1A), along with the expression of antioxidant-related factors, glutathione, and catalase. In the pentobarbital-induced sleep test using ICR mice, the duration of sleep was markedly increased by high concentration of DE. In addition, through the electroencephalography analysis of SD-rats, a significant increase in non-rapid-eye-movement sleep and delta waves was confirmed with high concentrations of DE administration. The increase in sleep time and improvement in sleep quality were confirmed to be related to the expression of altered GABA receptors and the enhancement of the contents of the neurotransmitters GABA and serotonin (5-HT) because of high DE administration. High-dose administration of DE also increased the expression of antioxidant-related factors in the brain and significantly decreased malondialdehyde content. Taken together, DE induced improvements in sleep quantity and quality by regulating neurotransmitter content and related receptor expression, along with high antioxidant activity, and may have a therapeutic effect on sleep disorders.

## 1. Introduction

The persistence and neglect of sleep disorders, including insomnia induced by genetic and external factors, are also related to the development of numerous diseases and inflammatory signaling pathways in the brain [1]. During wakefulness, substantial amounts of oxygen are used to maintain neuronal membrane potential, which subsequently generates cytotoxic reactive oxygen species (ROS). As a product of cell metabolism, ROS react with several important biomolecules, including nucleic acids, proteins, and membrane lipids to induce apoptosis and is reported as a cause of cognitive impairment and nerve damage in neuroscience [2]. Moreover, symptoms of sleep disorders are defined as complaints associated with daytime dysfunction, including reduced attention span, fatigue, exhaustion, discomfort, and other symptoms [3].

Sleep disorder treatment can be divided into cognitive–behavioral therapy and chronic or short-term medications, and pharmacological approaches typically include benzodiazepines (alprazolam, clonazepam, and lorazepam) and nonbenzodiazepines (zolpidem). Although both bind to gamma-aminobutyric acid (GABA) type A receptors and mediate rapid inhibitory synaptic transmission via chloride channel activation, the long-term use of pharmacological interventions has side effects, such as dependence, resistance, and reduction of memory [4]. Owing to the side effects of various reported pharmaceuticals, the evaluation and development of botanical extracts from plants, such as *Valeriana officinalis* [5], *Camellia sinensis* [6], *Withania somnifera* [7], *Curcuma longa* [8], and *Nelumbo nucifera* [9], which are weak in efficacy compared to constraints, is gradually increasing. These substances exhibit sleep-promoting effects via antioxidant activity in the brain as well as regulation of GABA, serotonin (5-HT), and histamine receptors using cell lines, specific neurotransmitter receptor binding assays, and animal models [10].

Metabolic consequences of sleep deprivation include antioxidant imbalances, indicating a bidirectional relationship between sleep and oxidative stress [11]. Disruption of sleep patterns reduces the activity of antioxidant enzymes and decreases physical and mental capacity to respond to oxidative stress, and elevated levels of oxidative stress markers have been observed in individuals with sleep disorders, such as insomnia or sleep apnea [12]. Oxidative-stress-induced inflammation and cellular damage can disrupt normal sleep structure and impair sleep quality, and activation of the inflammatory response causes alterations in neurotransmitter systems and disruption of circadian rhythms [2]. Maintaining healthy sleep patterns is essential for minimizing oxidative stress and promoting a balanced regular physical activity and nutritional aspect to reduce oxidative stress levels and improve sleep quality.

*Dendropanax morbiferus* is a plant belonging to the family *Araliaceae* and contains numerous phytochemical and bioactive compounds, such as rutin, chlorogenic acid, quercetin, and p-coumaric acid [13]. Previous experimental results have reported that *D. morbiferus* leaf extracts (DE) affect inflammatory diseases, breast cancer, periodontitis constipation, and oligodendrocyte development based on their excellent antioxidant activity [13,14,15]. In addition, DE has been observed to have anti-obesity and cholesterol-lowering activities, as well as efficacy against neuroinflammation and behavioral impediments [16,17,18]. DE was not reported until recently for quantitative or qualitative improvement in sleep, but the depressant action of rutin and chlorogenic acid, which are single components contained in the extract, on central nervous system was determined in animal models and clinical trials [19].

The purpose of this study using invertebrates and vertebrates was to confirm the improvement in sleep elevation time and sleep quality of DE-containing polyphenols such as rutin and chlorogenic acid through analysis of behavioral changes, total sleeping time, and brain wave pattern. In addition, by analyzing the content of neurotransmitters and the expression of sleep-related receptors, we aimed to report the mechanism related to sleep improvement according to DE administration.

## 2. Materials and Methods

### 2.1. Materials

Sodium hydroxide and propionic acid were purchased from Daejung Chemicals (Siheung, Republic of Korea). For pentobarbital, Entobar (100 mg) from Hanlim Pharmaceutical Co. (Yongin, Republic of Korea) and for benzodiazepine (BDZ), Xanax tablets (0.5 mg) from Pfizer Korea Ltd. (Seoul, Republic of Korea) were used. Chemicals not described were purchased from Sigma (Sigma Aldrich, St. Louis, MO, USA).

### 2.2. Plant Material and Preparation of Extracts

Dried *D. morbiferus* leaves were supplied by JEJUPANATEK (JEJUPANATEK Inc., Jeju, Republic of Korea). Ground *D. morbiferus* leaves (50 g) were added to 70% ethanol (500 mL, *w*:*v* = 1:10), followed by reflux extraction at 80 °C for 2 h. After repeating the extraction process once, the extract was filtered (Whatman No. 2, Whatman plc, Kent, UK) and concentrated under reduced pressure (R-100, BUCHI Labortechnik AG, Flawil, Switzerland). Extracts were lyophilized (FDTE-8012, Operon, Gimpo, Republic of Korea) and stored at −18 °C until further processing. The yield of the extract to the original mass was 24%.

### 2.3. Analysis of Antioxidant Capacity

The antioxidant capacity of DE was evaluated by 2,2’-azino-bis(3-ethylbenzothiazoline-6-sulfonic acid) (ABTS) and 1,1-diphenyl-2-picrylhydrazyl (DPPH) radical scavenging activities according to the method described previously [20]. The antioxidant capacity of DE was expressed as IC_50_, which is the concentration that reduces ABTS and DPPH radicals by 50%.

### 2.4. Analysis of Polyphenol Components

The polyphenolic composition in the DE was analyzed via high-performance liquid chromatography (Waters Scientific Ltd., Mississauga, ON, Canada) using a YMC-Triart C18 column (250 × 4.6 mm, 5 μm, YMC, Kyoto, Japan). The mobile phase was water and acetonitrile containing 0.2% formic acid, the flow rate was 0.8 mL/min, and the injection volume was 10 μL. The measurement wavelength was 260 nm for gallic acid, 3,4-dihydroxybenzoic acid, and rutin; 310 nm for chlorogenic acid, p-coumaric acid, caffeic acid, trans-ferulic acid, and apigenin; and 365 nm for quercetin and kaempferol [21].

### 2.5. Drosophila Melanogaster Stocks

Wild-type *Drosophila melanogaster* Canton-S strain from the Bloomington *Drosophila* Stock Center (Bloomington, IN, USA) was obtained. The incubator (HB302L, Hanback Co., Ltd., Buchun, Republic of Korea) maintained a temperature of 23 ± 1 °C and a humidity of 65 ± 5% and a light–dark cycle of 12:12. The standard media (sucrose, agar, cornmeal, dried yeast, propionic acid, and ρ–hydroxybenzoic acid methyl ester solution) were used in culture. Three-day-old male *Drosophila* were collected under CO_2_ anesthesia and used in the experiment.

### 2.6. Analysis of Sleep Behavior

The *Drosophila* activity monitoring (DAM) system was used to analyze the movement of fruit flies. Each fruit fly was placed in a transparent DAM tube (65 × 55 mm), and infrared rays were irradiated at the center of the glass bottle every minute. The flies were exposed to DE-containing sucrose–agar medium (5% sucrose, 3% agar) after confirming that circadian rhythms were normalized in the presence or absence of light. Sleep activity was evaluated by analyzing the sum of total movements (no. of counts), the number of times sleep is maintained for more than 5 min (sleep bouts), and the sum of total sleep time (sleep duration).

### 2.7. Locomotor Activity Analysis

*Drosophila* (male, 3 days old) were exposed to DE-containing sucrose–agar medium for 5 d, and then the flies were analyzed for 5 min by putting them one by one in nine circular arenas (8 mm in diameter, 1 mm in height). The EthoVision-XT system (Noldus Information Technology, Wageningen, The Netherlands) was used to analyze behavioral indicators of moving distance, velocity, moving, not moving, and mobility [22]. 

### 2.8. Analysis of Gene Expression via Quantitative Real-Time Polymerase Chain Reaction (qRT-PCR)

*Drosophila* (50 per group) was exposed to DE-containing sucrose–agar medium (1, 2, and 4%) for 7 days, and then the fly heads were collected. ICR mice were orally administered with DE (100 and 200 mg/kg) for 8 d, euthanized under CO_2_ anesthesia, and brains were collected. Total RNA was extracted from both *Drosophila* head and mouse brain with RNAzol reagent (Invitrogen, Carlsbad, CA, USA) according to previously reported methods [23], and cDNA was synthesized using Superscript III Reverse Transcriptase (Invitrogen). Target gene expression was analyzed using TaqMan PCR Master Mix (Applied Biosystems, Foster City, CA, USA), and ribosomal protein L32 (RpL32, NM_001144655.3) and glyceraldehyde-3-phosphate dehydrogenase (GAPDH, NM_001289726.1) were used as endogenous genes. The target genes used in the *Drosophila* model are as follows: GABA type A receptor (GABA_A_-R, NM_001274688.1), GABA type B receptor subunit 1 (GABA_B_-R1, NM_001259104.1), GABA type B receptor subunit 2 (GABA_B_-R2, NM_001300527.1), 5-hydroxytryptamine receptor 1A (5-HT1A, NM_166322.2), superoxide dismutase (SOD, NM_057387.5), catalase (CAT, NM_080483.3), and glutathione peroxidase (GPX, NM_168025.2). In addition, the following target genes were used in the mouse model: GABA_A_-R (NM_008076.3), GABA_B_-R1 (NM_019439.3), GABA_B_-R2 (NM_001081141.1), 5-hydroxytryptamine receptor 1A (Htr1a, NM_008308.4), SOD (NM_011434.2), CAT (NM_009804.2), and Gpx1 (NM_001329528.1).

### 2.9. Animals

Sprague-Dawley rats (5 weeks old, male) were purchased from Orient Bio (Seongnam, Republic of Korea), and ICR mice (3 weeks old, male) were purchased from Cronex (Seoul, Republic of Korea). The environment of the animal laboratory (temperature: 24 ± 1 °C, humidity: 50 ± 5%, 12-h day/night cycle) was maintained continuously, and water and feed were supplied ad libitum. All animal experiments were performed with the approval of the Institutional Animal Care and Use Committee of Jeju National University (approval number: 2022-0037, approval date: 22 August 2022).

### 2.10. Pentobarbital-Induced Sleep Test

Experimental animals were fasted for 20 h before the experiment after going through a 1-week adaptation period, and the experiment was conducted between 1:00 PM and 5:00 PM. Forty minutes after oral administration of DE (100 and 200 mg/kg) or benzodiazepine (BDZ, 0.2 mg/kg), pentobarbital (42 mg/kg) was intraperitoneally injected. After pentobarbital injection, all mice were moved to an independent space, and sleep latency and total sleep time were measured [7].

### 2.11. Electroencephalogram (EEG) Analysis

Electrode insertion surgery was performed 1 week before the experiment according to the previously reported method [7]. Samples (DE and BDZ) were administered orally at 9 AM, and EEG was recorded for 9 days at 15 mm/s for 7 h from 10:00 AM to 17:00 PM. Sleep structure analysis was performed using the Fast Fourier Transform algorithm and was calculated using the ecgAUTO3 program (Ver, 3.3, emka Technologies, Paris, Franca). EEG analysis results were divided into sleep, awake, rapid-eye-movement (REM) sleep, non-rapid-eye-movement (NREM) sleep, θ wave, and δ wave to the frequency (γ: 30–60 Hz; β: 12–30 Hz; α: 8–12 Hz; θ: 4–8 Hz; δ: 0.5–4 Hz) [9].

### 2.12. Enzyme-Linked Immunosorbent Assay (ELISA)

GABA and 5-HT contents were measured in the mouse brain using ELISA. The samples were orally administered for 8 d and euthanized under CO_2_ anesthesia to collect brains. The brain used in the experiment was stored at −80 °C until the experiment. After homogenization, the brain was analyzed using the ELISA kit (MyBioSource Inc., San Diego, CA, USA). The experiment was carried out according to the manual enclosed with the kit. After measuring total protein via BCA analysis, the value was corrected. The kits used were Mouse Gamma Aminobutyric Acid ELISA Kit (#MBS725233) and Mouse Serotonin ST ELISA Kit (#MBS1601042).

### 2.13. Malondialdehyde (MDA) Assay

The rat brain tissue supernatant used for measuring the MDA content was the same as that used for ELISA analysis. MDA content was measured using the OxiTec TBARS Assay Kit (Biomax Co, Ltd., Seoul, Republic of Korea) product and according to the accompanying instructions. Values were corrected after measuring the total protein content using BCA assay.

### 2.14. Statistical Analysis

Data are presented as mean ± standard error of the mean (SEM) and means ± standard deviation (SD). Statistical analysis was assessed using Prism (8.0.1., GraphPad Software Inc., San Diego, CA, USA). To compare them, the groups of data were analyzed via Tukey’s multiple-range test and Student’s *t*-test using the statistical package for social science. Statistical significance was set at *p* < 0.05.

## 3. Results

### 3.1. Antioxidant Activity and Polyphenol Components of DE

DE scavenged 50% of ABTS radicals at a concentration of 0.70 ± 0.02 mg/mL and 50% of DPPH radicals at a concentration of 0.32 ± 0.02 mg/mL (Table 1). In addition, rutin and chlorogenic acid scavenged 50% of ABTS radicals at a concentration of 0.23 ± 0.01 mg/mL and 0.11 ± 0.01 mg/mL, and 50% of DPPH radicals at a concentration of 0.11 ± 0.01 mg/mL and 0.08 ± 0.00 mg/mL, respectively (Table 1). The IC_50_ of DE was 1/10 of that of ascorbic acid used as a standard. Figure S1 shows the HPLC chromatograms of the standard and DE. As a result of analyzing the flavonoid composition of DE (Table 2), the contents of chlorogenic acid and rutin were 59.22 μg/mg and 35.78 μg/mg, respectively. Apigenin and kaempferol were not detected in the DE, and the total polyphenol content of DE was 98.88 μg/mg.

### 3.2. Evaluation of Sleep Activity of DE in Drosophila

**Figure 1:**
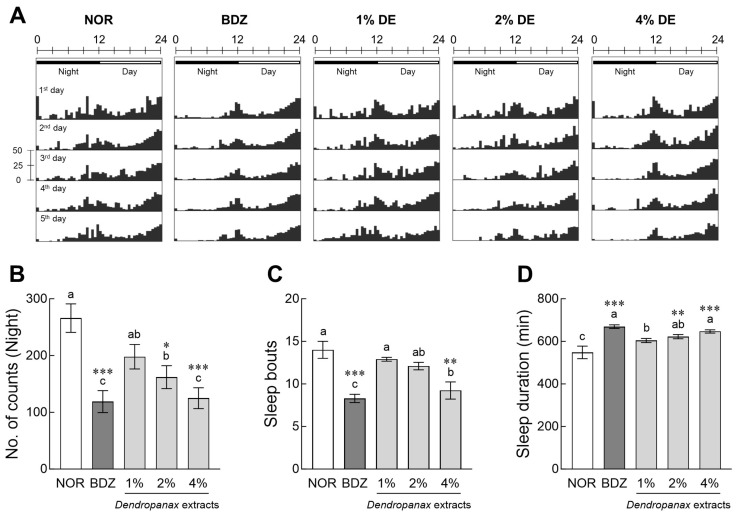
Effects of *Dendropanax morbiferus* extract (DE) on sleep activity in *Drosophila melanogaster*. Behavioral analysis was performed after an adaptation period under the light for 1 day and then turning off the light for 5 days. In the actogram, the upper black bar indicates the nighttime phase (PM 10:01 to AM 10:00), and the white bar indicates the daytime phase (AM 10:01 to PM 10:00). (**A**) Actogram, (**B**) subjective nighttime activity, (**C**) number of sleep episodes, and (**D**) subjective nighttime sleep duration in the DAM. Data are presented as the mean ± the standard error of the mean (SEM) for each group. Different letters indicate significant differences at *p* < 0.05 using Tukey’s test, * *p* < 0.05, ** *p* < 0.01, and *** *p* < 0.001 vs. NOR. NOR, normal group; BDZ, positive control group.

The movements of *Drosophila* at night (10:01 PM to 10:00 AM, black bar) and daytime (10:01 AM to 10:00 PM, white bar) were visualized as actograms (Figure 1A). The BDZ, positive control, and DE-treated flies showed a tendency to decrease nighttime movement compared to the normal group (NOR) depending on the administration period. Treatment with 2% and 4% DE significantly reduced the total activities of subjective nighttime activities of flies compared to the NOR by 31% (*p* < 0.05) and 53% (*p* < 0.001), respectively (Figure 1B). In addition, 0.1% BDZ and 4% DE treatment significantly reduced the number of sleep bouts compared to NOR, improving sleep-to-sleep interruptions (*p* < 0.001 and *p* < 0.01, respectively; Figure 1C). The DE treatment group increased the total sleep time in a dose-dependent manner (Figure 1D), and in particular, the 2% and 4% DE treatment groups showed significantly higher total sleep time than the NOR group (*p* < 0.01 and *p* < 0.001, respectively). In Figure S2, when analyzing trend changes for five consecutive days, BDZ showed immediate effects of sedative on sleep activities in *Drosophila*, and DE treatments were delayed but stabilized.

### 3.3. Effects of DE on Locomotor Activity in Drosophila

**Figure 2:**
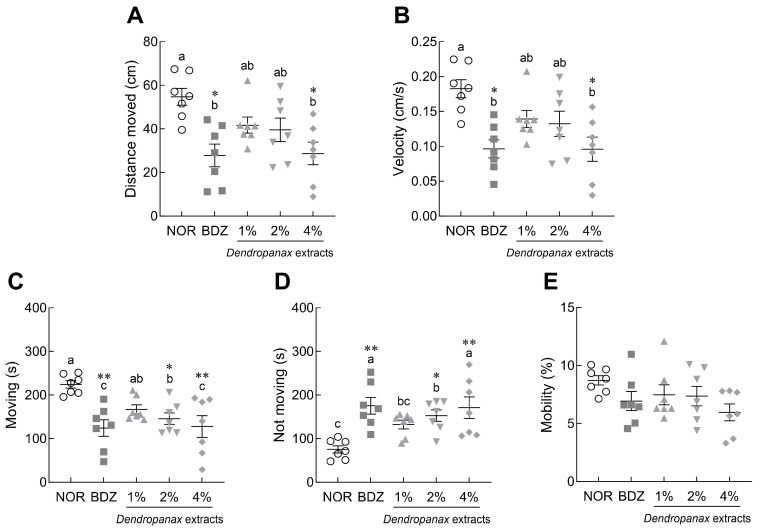
Effects of *Dendropanax morbiferus* extract (DE) on (**A**) distance moved, (**B**) velocity, (**C**) moving, (**D**) not moving, and (**E**) mobility in *Drosophila melanogaster*. After 5 days of exposure, the locomotion during the 5 min observation period in the video tracking was analyzed using the EthoVision-XT system. Values are the means ± standard error of mean (SEM) for each group. Different letters indicate significant differences at *p* < 0.05 using Tukey’s test, * *p* < 0.05, ** *p* < 0.01 vs. NOR. NOR, normal group; BDZ, positive control group.

Behavioral indicators were analyzed in *Drosophila* after 5 days of DE treatment (Figure 2). DE (4%) and BDZ (0.1%) treatments significantly reduced the distance moved (*p* < 0.05; Figure 2A) and velocity, respectively (*p* < 0.05; Figure 2B), in *Drosophila* compared to the NOR group. The 2% and 4% DE treatment and BDZ groups showed a significant decrease in total moving time compared to the NOR group (*p* < 0.05 and *p* < 0.01, respectively; Figure 2C), whereas the non-moving time significantly increased (*p* < 0.05 and *p* < 0.01, respectively; Figure 2D). The mobility of flies tended to decrease in the DE-treated group compared to the NOR group, but there was no significant difference (Figure 2E).

### 3.4. Effect of DE on mRNA Expression of Sleep-Related Receptors in Drosophila

**Figure 3:**
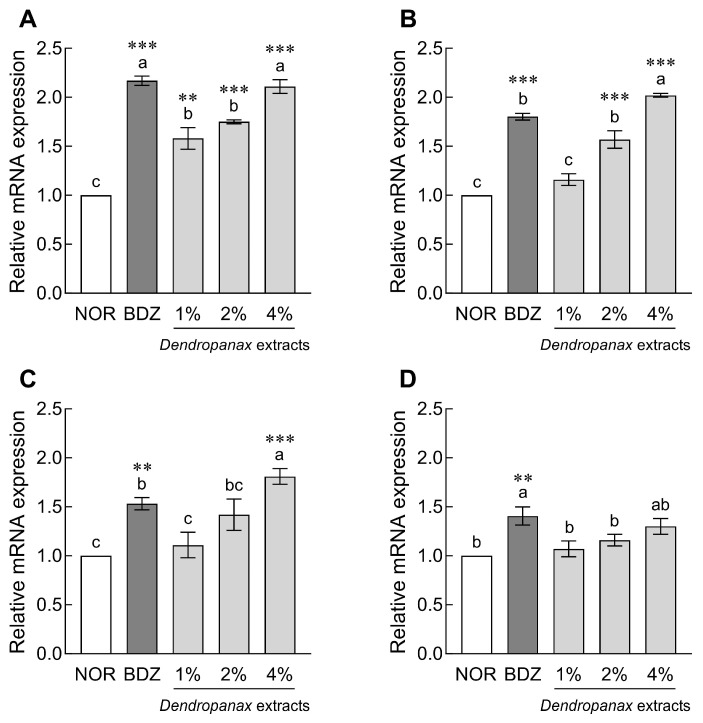
Effects of *Dendropanax morbiferus* extract (DE) on mRNA expression associated with sleep of *Drosophila melanogaster*. (**A**) GABA_A_-R, (**B**) GABA_B_-R1, (**C**) GABA_B_-R2, and (**D**) 5-HT1A. Values are the means ± standard error of the mean (SEM) for each group. Different letters indicate significant differences at *p* < 0.05 using Tukey’s test, ** *p* < 0.01, and *** *p* < 0.001 vs. NOR. NOR, normal group; BDZ, positive control group; GABA_A_-R, gamma-aminobutyric acid type A receptor; GABA_B_-R1, gamma-aminobutyric acid type B receptor subunit 1; GABA_B_-R2, gamma-aminobutyric acid type B receptor subunit 2; 5-HT1A, 5-hydroxytryptamine receptor 1A.

The mRNA expression levels of GABA and 5-HT receptors in *Drosophila* heads are shown in Figure 3. DE (1%, 2%, and 4%) treatment significantly increased the mRNA expression of GABA_A_-R compared to the NOR group in a dose-dependent manner (*p* < 0.01 and *p* < 0.001, respectively; Figure 3A). In addition, treatment with 2% and 4% DE significantly increased GABA_B_-R1 expression 1.57-fold and 2.02-fold, respectively, compared to the NOR group (*p* < 0.001; Figure 3B). In particular, the 4% DE treatment group showed significantly higher GABA_B_-R2 (1.81-fold, *p* < 0.001; Figure 3C) and 5-HT1A (1.30-fold, *p* < 0.05; Figure 3D) contents than the NOR group. In the case of the BDZ group, which was a positive control, the expression of GABA_A_-R, GABA_B_-R1, GABA_B_-R2, and 5-HT1A receptors was significantly increased compared to the NOR group (*p* < 0.001 and *p* < 0.01; Figure 3).

### 3.5. Effects of DE on Sleep Activity and Antioxidant Enzymes Expression in Insomnia-Induced Drosophila Model

**Figure 4:**
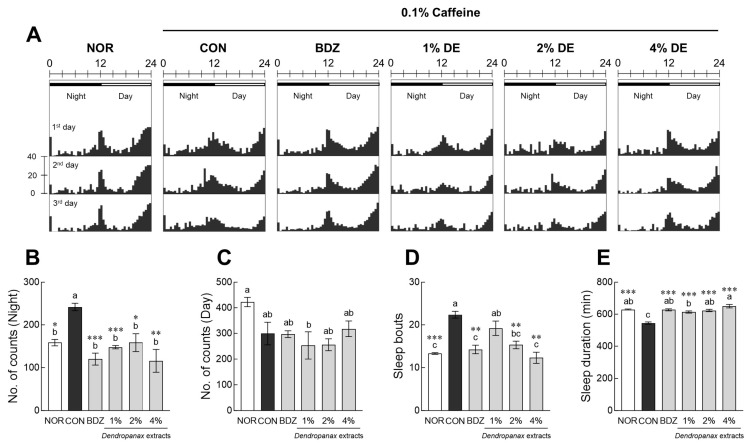
Effects of *Dendropanax morbiferus* extract (DE) on locomotor activity in caffeine-induced *Drosophila melanogaster* insomnia model. Behavioral analysis was performed after having an adaptation period under the light for 1 day and then turning off the light for 3 days. In the actogram, the upper black bar indicates the nighttime phase (PM 10:01 to AM 10:00), and the white bar indicates the daytime phase (AM 10:01 to PM 10:00). (**A**) Actogram, (**B**) subjective nighttime activity, (**C**) subjective daytime activity, (**D**) number of sleep episodes, and (**E**) subjective nighttime sleep duration. Data are presented as the mean ± the standard error of the mean (SEM) for each group. Different letters indicate significant differences at *p* < 0.05 using Tukey’s test. * *p* < 0.05, ** *p* < 0.01, and *** *p* < 0.001 vs. CON using Student’s *t*-test. NOR, normal group; CON, caffeine-control group; BDZ, positive control group.

To induce insomnia, *Drosophila* was cultured in sucrose–agar medium containing 0.1% caffeine. In the actogram (Figure 4A), the caffeine-control (CON) group tended to increase subjective night activity and decrease daytime activity compared to the NOR group. Subjective nighttime activity in the CON group showed a significant increase compared to that in the NOR group (1.52-fold, *p* < 0.05; Figure 4B). BDZ and DE treatment significantly ameliorated nighttime activity increased by caffeine at all concentrations (*p* < 0.05, *p* < 0.01 and *p* < 0.001, respectively; Figure 4B). Subjective daytime activity tended to decrease in the CON group compared to that in the NOR group, but no significant difference was noted between the positive control (0.1% BDZ) and experimental groups (Figure 4C). Sleep bouts in the CON group were significantly higher than those in the NOR group (*p* < 0.001; Figure 4D). However, BDZ and DE (2% and 4%) treatments significantly reduced sleep bouts compared to the CON group (*p* < 0.01, respectively; Figure 4D). Caffeine treatment significantly reduced the total sleep time by 0.86 times compared to the NOR group (*p* < 0.001; Figure 4E). DE (1%, 2%, and 4%) and BDZ treatment significantly improved the total sleep time reduced by caffeine in a dose-dependent manner (*p* < 0.001).

**Figure 5:**
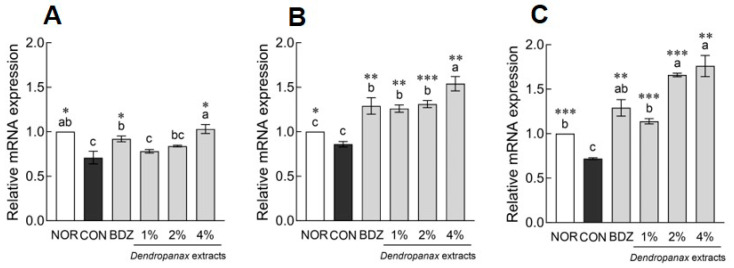
Effects of *Dendropanax morbiferus* extract (DE) on mRNA expression associated with antioxidant activity of *Drosophila melanogaster*. (**A**) SOD, (**B**) GPX, and (**C**) CAT. Values are the means ± standard error of the mean (SEM) for each group. Different letters indicate significant differences at *p* < 0.05 by Tukey’s test. * *p* < 0.05, ** *p* < 0.01, and *** *p* < 0.001 vs. CON using Student’s *t*-test. NOR, normal group; CON, caffeine-control group; BDZ, positive control group; SOD, superoxide dismutase; GPX, glutathione peroxidase; CAT, catalase.

The effect of DE on the expression of antioxidant enzymes in the caffeine-treated *Drosophila* model is shown in Figure 5. Caffeine treatment significantly reduced the mRNA expression of antioxidant enzymes, such as SOD (*p* < 0.05; Figure 5A), GPX (*p* < 0.01; Figure 5B), and CAT (*p* < 0.01; Figure 5C), compared to the NOR group. DE treatment (4%: *p* < 0.05) significantly increased the mRNA expression of SOD in a dose-dependent manner compared to the CON group (Figure 5A). Similarly, the DE treatment group significantly improved the reduction of GPX (1%: *p* < 0.01, 2%: *p* < 0.001, and 4% *p* < 0.01; Figure 5B) and CAT (1%: *p* < 0.001, 2%: *p* < 0.001, and 4%: *p* < 0.01; Figure 5C) expression by caffeine in a dose-dependent manner. The BDZ group, a positive control group, significantly increased the expression of antioxidant-related genes SOD, GPX, and CAT compared to the CON group (*p* < 0.05 and *p* < 0.01; Figure 5). In addition, when normal *Drosophila* were treated with DE, mRNA expression of antioxidant activity-related genes tended to increase in a concentration-dependent manner, but there was no significant difference, confirming that DE treatment for a short period of time did not affect gene expression (Figure S3).

### 3.6. Pentobarbital-Induced Sleep Test

**Figure 6:**
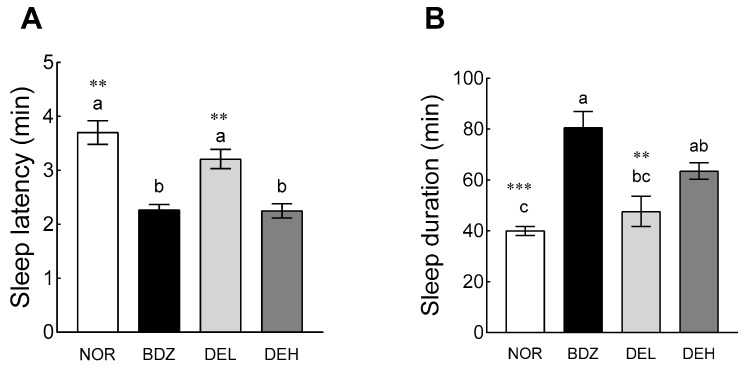
Effects of *Dendropanax morbiferus* extract (DE) on (**A**) sleep latency time and (**B**) sleep duration in ICR mice intraperitoneally administered pentobarbital (42 mg/kg). Values are the means ± standard error of the mean (SEM) for each group. Different letters indicate significant differences at *p* < 0.05 by Tukey’s test. ** *p* < 0.01 and *** *p* < 0.001 vs. BDZ using Student’s *t*-test. NOR—normal, 0.9% saline; BDZ—benzodiazepine, 0.2 mg/kg; DEL—*Dendropanax morbiferus* extract, 100 mg/kg; and DEH—*Dendropanax morbiferus* extract, 200 mg/kg.

The sleep-enhancing effect of DE was confirmed through a pentobarbital-induced sleep experiment (Figure 6). In sleep latency, the BDZ group showed a significant decrease in sleep latency compared to the NOR group (*p* < 0.05, Figure 6A). DE administration decreased sleep latency in a dose-dependent manner, and high-dose DE (DEH) showed significantly lower sleep latency than the NOR group (*p* < 0.05). In particular, the DEH group reduced sleep latency to a level similar to that of the BDZ group. In sleep time, the BDZ group (80.60 ± 6.34 min) showed a significant increase compared to the NOR group (40.00 ± 1.79 min) (*p* < 0.001; Figure 6B). High-dose DE administration (63.57 ± 3.21 min) also showed a significantly higher sleep time than the NOR group (*p* < 0.05).

### 3.7. EEG Pattern Recording

**Figure 7:**
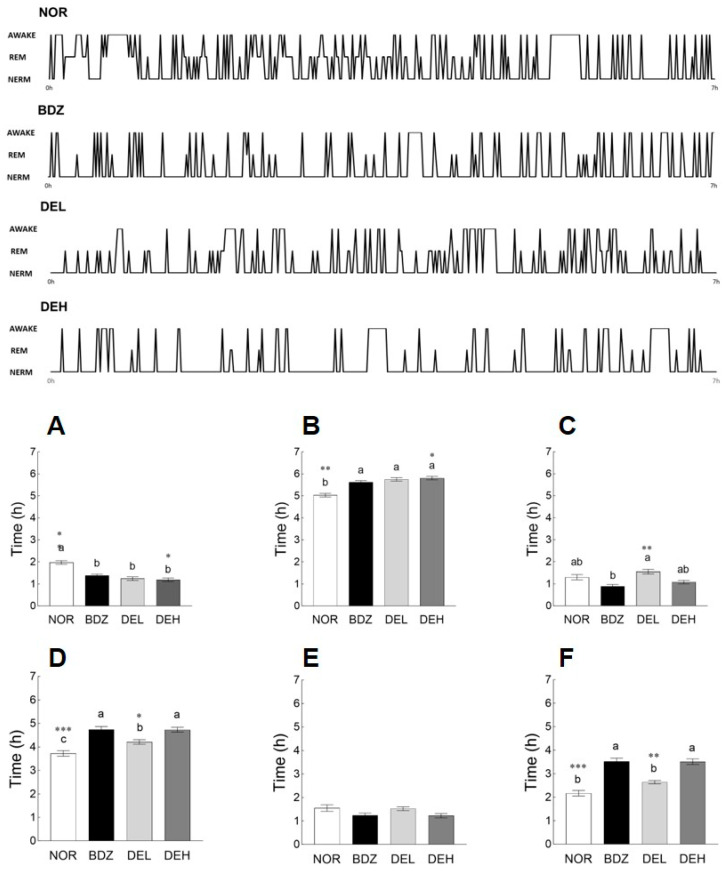
Effects of *Dendropanax morbiferus* extract (DE) on (**A**) awake, (**B**) sleep, (**C**) REM, (**D**) NREM, (**E**) θ sleep pattern, and (**F**) δ sleep pattern changed in rats. EEG analyses were conducted for 9 days, and DE was administered orally. Values are the means ± standard error of the mean (SEM) for each group. Different letters indicate significant differences at *p* < 0.05 by Tukey’s test. * *p* < 0.05, ** *p* < 0.01, and *** *p* < 0.001 vs. BDZ by Student’s *t*-test. NOR—normal, 0.9% saline; BDZ—benzodiazepine, 0.2 mg/kg; DEL—*Dendropanax morbiferus* extract, 100 mg/kg; and DEH—*Dendropanax morbiferus* extract 200 mg/kg.

An EEG was performed to evaluate sleep patterns and sleep quality. BDZ and DE (100 and 200 mg/kg) administration significantly decreased the awake time compared to the NOR group (*p* < 0.05; Figure 7A). In addition, administration of BDZ and DE significantly increased sleep time (*p* < 0.05; Figure 7B). In particular, administration of 200 mg/kg DE effectively reduced wake time and increased sleep time compared to BDZ. REM sleep tended to decrease in the BDZ and DEH groups than that in the NOR group, but no significant difference was observed (Figure 7C). NREM sleep was significantly increased by BDZ administration compared to that in the NOR group (*p* < 0.001, Figure 7D). Moreover, DE administration significantly increased NREM sleep compared to the NOR group in a dose-dependent manner (*p* < 0.05, Figure 7D). No significant difference was observed between all the experimental groups in the θ wave (Figure 7E). In the δ wave, the BDZ group showed a significant increase compared to the NOR group (*p* < 0.001; Figure 7F), and the DE administration showed a dose-dependent increase (*p* < 0.05). NREM and δ waves involved in deep sleep were improved by high-dose DE administration, which was similar to that of the BDZ group.

### 3.8. Effects of DE on mRNA Levels and Neurotransmitter Content in ICR Mice

**Figure 8:**
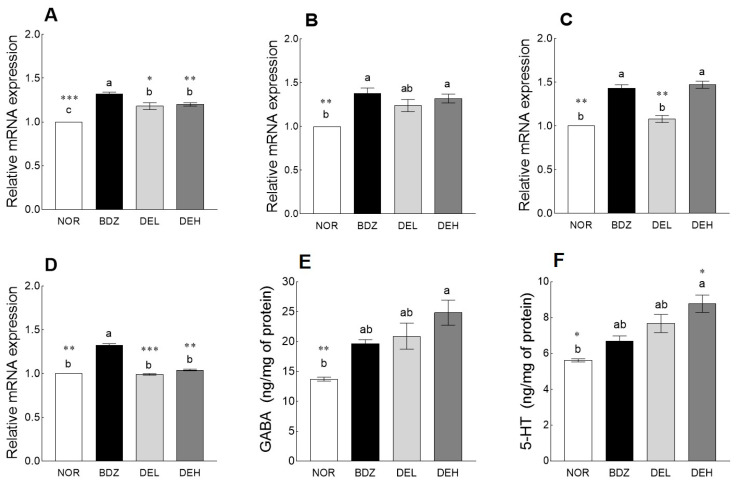
Effects of *Dendropanax morbiferus* extract (DE) on gene expression and neurotransmitter levels in sleep ICR mouse model. (**A**) GABA_A_-R, (**B**) GABA_B_-R1, (**C**) GABA_B_-R2, (**D**) Htr1a, (**E**) GABA, and (**F**) 5-HT. Values are the means ± standard error of the mean (SEM) for each group. Different letters indicate significant differences at *p* < 0.05 using Tukey’s test. * *p* < 0.05, ** *p* < 0.01, and *** *p* < 0.001 vs. BDZ using Student’s *t*-test. NOR—normal, 0.9% saline; BDZ—benzodiazepine, 0.2 mg/kg; DEL—*Dendropanax morbiferus* extract, 100 mg/kg; and DEH—*Dendropanax morbiferus* extract, 200 mg/kg. GABA_B_-R1, gamma-aminobutyric acid type B receptor subunit 1; GABA_B_-R2, gamma-aminobutyric acid type B receptor subunit 2; Htr1a, 5-hydroxytryptamine receptor 1A; GABA, gamma-aminobutyric acid; 5-HT, 5- hydroxytryptamine.

The effect of DE on sleep-related receptors was measured in the mouse brain via qRT-PCR (Figure 8A–D). BDZ administration significantly upregulated the mRNA expression of GABA_A_-R (1.32-fold, *p* < 0.001, Figure 8A), GABA_B_-R1 (1.38-fold, *p* < 0.01, Figure 8B), and GABA_B_-R2 (1.43-fold, *p* < 0.01, Figure 8C) compared to the NOR group. DE administration significantly upregulated the expression of GABA_A_-R compared to the NOR group (*p* < 0.05), and the high-dose DE administration group showed significantly higher expressions of GABA_B_-R1 (1.32-fold) and GABA_B_-R2 (1.47-fold) than the NOR group (*p* < 0.05). In addition, the BDZ group showed a significant increase in Htr1a expression compared to the NOR group (*p* < 0.01, Figure 8D), but no increase in Htr1a expression was observed through DE administration. As a result of sleep-related neurotransmitter analysis, DE administration increased the contents of GABA (Figure 8E) and 5-HT (Figure 8F) in a dose-dependent manner compared to the NOR group, which was higher than that of the BDZ group. In particular, 200 mg/kg DE administration significantly increased GABA (1.80-fold, *p* < 0.05) and 5-HT (1.56-fold, *p* < 0.05) contents compared to the NOR group.

### 3.9. Effects of DE on MDA Content and Antioxidant Enzyme-Related mRNA Expression in ICR Mice

**Figure 9:**
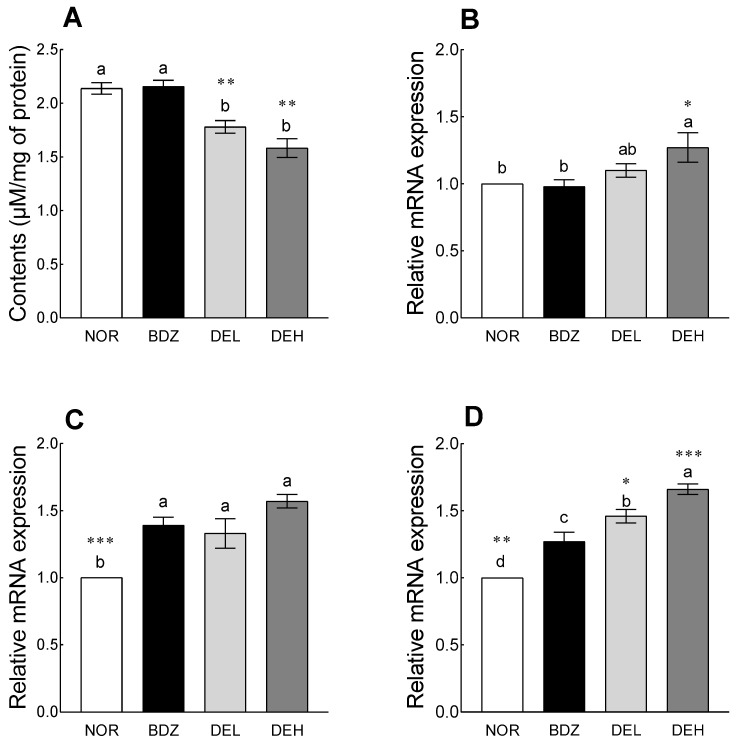
Effects of *Dendropanax morbiferus* extract (DE) on (**A**) MDA contents and gene expression of (**B**) SOD, (**C**) Gpx1, and (**D**) CAT in ICR mouse model. Values are the means ± standard error of the mean (SEM) for each group. Different letters indicate significant differences at *p* < 0.05 using Tukey’s test. * *p* < 0.05, ** *p* < 0.01, and *** *p* < 0.001 vs. BDZ using Student’s *t*-test. NOR—normal, 0.9% saline; BDZ—benzodiazepine, 0.2 mg/kg; DEL—*Dendropanax morbiferus* extract, 100 mg/kg; and DEH—*Dendropanax morbiferus* extract, 200 mg/kg. MDA, malondialdehyde; SOD, superoxide dismutase; GPX, glutathione peroxidase; CAT, catalase.

Compared to the NOR group, DE administration significantly reduced the levels of MDA in the brain, a representative superoxide produced by organisms and an indicator of oxidative stress, although the BDZ-administered group did not show a significant difference from the NOR group (Figure 9A, *p* < 0.01). SOD expression showed a tendency to increase in the DEL group compared to the NOR group and was significantly increased in the DEH group (Figure 9B, *p* < 0.05). In the case of the expression of Gpx1, all groups showed a significant increase compared to the NOR group, and compared to the NOR group, the BDZ group increased by 39%, DEL by 33%, and DEH by 57% (Figure 9C). The expression of CAT increased in all groups compared to the NOR group; the DEL group increased by 46% compared to the NOR group, and the DEH group increased by 66% (Figure 9D). Additionally, compared to the BDZ group, the DEL and DEH groups showed a significant increase (*p* < 0.05 and *p* < 0.001, respectively).

## 4. Discussion

A prevalent sleep disorder affects a substantial proportion of the general population, and the Diagnostic and Statistical Manual of Mental Disorders, Fourth Edition (DSM-IV) defines insomnia as the presence of one or more symptoms related to sleep disturbances in approximately one-third of the population [24]. Although sleep plays a vital role in the elimination of cytotoxic reactive species generated during the wakeful period, preventing oxidative stress within the physical condition, previous results suggest that ROS can modulate the activity of phasic and tonic GABA_A_ receptors and GABA release from presynaptic terminals [25]. Bioactive compounds contained in botanical extracts have been reported to promote sleep and alleviate symptoms of insomnia and may also indirectly contribute to sleep improvement and overall biological rhythm by reducing oxidative stress and scavenging ROS. Although recent evidence suggests the effects of *D. morbiferus*, which contains phytochemicals, on the molecular and morphological aspects of various diseases, there have been no reports of results related to the sleep-related efficacy of *D. morbiferus* extract.

Polyphenols, a class of bioactive compounds in various plant-based materials, have been identified as substances that cross the blood–brain barrier and have been reported for their potential effects on sleep quality, mental health, depression, and stress [26]. In our results, the polyphenol content of DE with high antioxidant capacity was approximately 98 μg/mg, of which the contents of rutin and chlorogenic acid accounted for approximately 96% of the total content (Table 1 and Table 2). Similarly, Eom et al. [27] reported that the main phenolic compounds in the 70% ethanol extract of *D. morbifera* leaves are chlorogenic acid (34.33 mg/g) and rutin (91.93 mg/g), and that they inhibit ethanol-induced ROS production through antioxidant activity. Additionally, the methanol extract of *D. morbifera* leaves contains various phenolic compounds such as rutin (23.7 μg/g), chlorogenic acid (16.1 μg/g), quercetin (5.3 μg/g), and p-coumaric acid (4.3 μg/g), and effectively inhibited lipopolysaccharide-induced inflammation in Raw 264.7 cells [16]. Rutin is a glycoside composed of flavonolic aglycone quercetin along with rutinose and is known to possess antioxidant, cytoprotective, and neuroprotective properties [28]. Chlorogenic acid, a water-soluble polyphenolic phenylacrylate compound, has been reported to be involved in biological activities, such as antioxidant, anti-inflammatory, glucose and lipid metabolism regulation, and nervous system protection [29,30].

In the present study, we used fruit flies to investigate changes in sleep-related parameters and locomotor activity following dose dependence of DE exposure (Figure 1 and Figure 2) and presented that the altered sleep-related behaviors depended on changes in GABA-related receptors and 5-HT1A expression (Figure 3). The *Drosophila* model has been used to analyze sleep-regulation-related mechanisms via molecular biological approaches, such as the GAL4-UAS system, as well as to analyze the sleep-promoting effects of botanical extracts, amino acids, and food materials [31]. Holvoet et al. assessed the effects of *Withania somnifera* (ashwagandha) ethanolic extract on sleep bouts and total sleeping time in aged flies and reported withanolides, which contained higher levels in ethanol extract than in water extract, as an active ingredient for sleep improvement [32]. High levels of β-acid and xanthohumol contents in Saphir, a hop (*Humulus lupulus* L.) variety, regulate sleep-related behaviors, including sleep duration by regulating GABAergic signaling [33]. GABA_A_ receptors, ionotropic receptors of GABA, mediate rapid inhibitory neurotransmission through ligand-gated chloride, and GABA_B_ receptors, metabotropic receptors, modulate and regulate the mid-term and terminal stages of sleep over a more extended period [34]. In addition, serotonin promotes baseline sleep in *Drosophila*, and it is known that the regulation of sleep behavior is linked to specific receptors in the brain [35]. Based on our mRNA-expression-related results, it was demonstrated that the increase in the expression of the two neurotransmitter-related receptors has immediate and stable sleep-promoting effects.

Using a caffeine-induced sleepless model, we investigated that DE exposure alleviated behavior altered by 0.1% caffeine (Figure 4) and enhanced the mRNA expression of antioxidant enzyme-related genes in the brain (Figure 5). Caffeine induces arousal by blocking adenosine A2A receptors and regulating protein kinase A and cAMP and is also a chemical widely used to construct an insomnia model in *Drosophila* [36]. In the case of Sansoninto, a Japanese traditional herbal medicine, improvement in caffeine-induced insomnia duration in a *Drosophila* model has been reported using an automated sleep and rhythm analysis system, and the sleep-enhancing effect of the material has been found to be related to activation of the GABAergic system and serotonergic system [37,38]. Our previous study reported that a mixture of 5-hydroxytryptophan, a precursor 5-HT, and GABA induces total sleeping time and increases NREM sleep in caffeine-mediated sleep loss in a vertebrate model linked to a *Drosophila* model [39]. Although exposure to 0.016 mM caffeine in a *Drosophila* mutant line lacking PGRP-LB with hyperactivation of NF-κB and 0.05% caffeine in wild type showed a positive effect on the endogenous antioxidant genes and enzymes, the number of nighttime activities and total sleep time were significantly reduced with decreased gene expression of SOD and CAT at 0.1% caffeine concentration [40,41].

According to the results obtained in pentobarbital-induced sleep test and EEG signal analysis, DE administration increased NREM sleep and related EEG, which correspond to sleep quality, along with the total sleep time (Figure 6 and Figure 7). The effect of DE on the quantity and quality of sleep has not been reported, but studies on the function of each major polyphenol contained have been steadily conducted. Fernández et al. analyzed the effect of rutin on sleep time and sedative action through thiopental-induced sleep test and behavioral analysis and reported on the possibility of use as a sleep-promoting material [42]. Previous studies have shown that subacute ingestion of chlorogenic acids decreased sleep latency in clinical trials [43], and in vitro and in vivo studies have reported that it prevents cognitive decline and nerve damage through tumor necrosis factor (TNF) and nuclear factor erythroid 2 related factor 2 (Nrf2)-nuclear factor kappa-light-chain-enhancer of activated B (NF-κB) signaling pathways [44,45].

The neurotransmitters GABA and 5-HT, which are associated with sleep, and related receptors significantly increased in the DE-administered group compared with that in the normal group (Figure 8). Animal studies and epidemiological evidence suggest that polyphenol administration may correlate with the promotion of sleep and sedation through regulation of specific neurotransmitter receptors as well as reduced oxidative stress and neuroprotection [46,47]. The anti-anxiety effect of the microinjected rutin in the basal amygdala regulated by GABA_A_/BDZ receptors as a major brain region was analyzed through the elevated plus-maze and open-field tests, and using flumazenil and picrotoxin, which are antagonists of BDZ and chloride channel GABA_A_, rutin can reduce anxiety through GABAergic neurotransmission [48]. Through analysis of the mechanism of action using flumazenil and WAY 100635, antagonists of GABA/BZD and 5-HT1A receptors, respectively, the anxiolytic and sedative-like effects of rutin and isoquertin, glycosides of quercetin contained in *Tilia americana* var. *mexicana*, were reported [49]. Moreover, 14 days of treatment with rutin in rats induced with reserpine-induced anxiety and depression showed antidepressant properties by increasing 5-HT content in cortical and hippocampal regions [50]. In the case of *Hypericum origanifolium* extract containing rutin and chlorogenic acids as major phenolic compounds, significant antidepressant and anxiolytic activity was induced by affecting the GABA_A_-benzodiazepine receptor complex after acute administration [51]. When evaluating the quantity and quality of sleep in rats, compared to caffeine, chlorogenic acid showed a mild arousal effect through its metabolite, caffeic acid, but did not cause significant changes in sleep status [52]. Clinical trials have shown that continuous consumption of an active beverage containing 300 mg of chlorogenic acid for 13 days positively affects fatigue upon awakening and sleep quality, while 600 mg of chlorogenic acid does not adversely affect sleep quality [43,53]. Wu et al. reported that feeding young pigs with chlorogenic-acid-containing feed induced changes in the levels of gut microbiota, 5-HT, free amino acids, and colonic 5-HT, which could affect brain function via the vagus nerve and blood circulation [54]. In addition, chlorogenic-acid-enriched extract from *Eucommia ulmoides* Oliver is known to be involved in neuromodulation by regulating synapsin I expression across the blood–cerebral fluid barrier and promoting 5-HT release [55].

We analyzed changes in oxidative stress markers, such as ROS production and gene expression after DE treatment in a mouse model (Figure 9). Sleep deprivation induces oxidative stress through ROS accumulation and in severe cases affects lifespan. When oxidative-stress-related gingival crevicular fluid levels were measured in school-aged children and teenagers, it was confirmed that MDA and H_2_O_2_ were substantially higher in sleep-deprived teenagers, whereas glutathione (GSH) was substantially decreased [56]. Previous studies have reported that sleep deprivation decreases the level of GSH in the hypothalamus and thalamus, possibly contributing to functional deficits [57]. Further studies on molecular approaches are needed to determine whether sleep deprivation causes biochemical abnormalities, including cellular oxidative damage. Results from numerous in vitro and in vivo studies have showed that rutin is involved in neuroprotective effects through antioxidant activity and activation of brain-derived neurotrophic factor and mitogen-activated protein kinase cascades [58]. In addition, intestinal microorganisms change through chlorogenic acid regulate intestinal inflammation and can modulate the biological function of the gut–brain axis involved in neurotransmitter secretion [59].

## 5. Conclusions

Taken together, DE-containing polyphenol, such as rutin and chlorogenic acid, induced an increase in total sleep time and a decrease in behavior through the expression of GABAergic and serotonergic signaling-related receptors and antioxidant-related enzymes in the fruit fly model. Through mouse and rat models, we confirmed that DE treatment improved sleep quality by significantly increasing NREM sleep and delta waves and found that these results are related to changes in neurotransmitters and associated receptors and antioxidant capacity of the material. Our results demonstrated the potential of plant-derived extracts containing rutin and chlorogenic acid to increase the quantitative aspects of sleep by regulating neurotransmission and reducing oxidative stress generated in the brain.

## Figures and Tables

**Table 1:** Antioxidant capacity of *Dendropanax morbiferus* extract (DE).

	ABTS (IC_50_, mg/mL)	DPPH (IC_50_, mg/mL)
*D. morbiferus* extract	0.70 ± 0.02	0.32 ± 0.02
Rutin	0.23 ± 0.01	0.11 ± 0.01
Chlorogenic acid	0.13 ± 0.00	0.08 ± 0.00

Values are the means ± standard deviation (SD) for each group.

**Table 2:** Active compounds of *Dendropanax morbiferus* extract (DE).

Compound	Gallic Acid	3,4-Dihydroxybenzoic Acid	Rutin	Chlorogenic Acid	Caffeic Acid	p-Coumaric Acid	Trans Ferulic Acid	Quercetin	Total Polyphenol
Contents (μg/mg)	0.15 ± 0.00	2.64 ± 0.00	35.78 ± 0.01	59.22 ± 0.02	0.43 ± 0.01	0.36 ± 0.00	0.12 ± 0.00	0.17 ± 0.00	98.88 ± 0.03

Values are the means ± standard deviation (SD) for each group.

## Data Availability

The data presented in this study are available on request from the corresponding author.

## Supplementary Materials

The following supporting information can be downloaded at: https://www.mdpi.com/article/10.3390/antiox12101890/s1, Figure S1. Chromatograms of polyphenols standard and sample using HPLC analysis. A: polyphenols standard, B: gallic acid, 3,4-dihydroxybenzoic acid, and rutin were measured at 260 nm, C: chlorogenic acid, p-coumaric acid, caffeic acid, trans-ferulic acid, and apigenin were measured at 310 nm, D: quercetin and kaempferol were measured at 365 nm.; Figure S2. Effects of *Dendropanax morbiferus* extract (DE) on the trend change of sleep activity in Drosophila melanogaster over 5 consecutive days. Behavioral analysis was performed after an adaptation period under the light for 1 day and then turning off the light for 5 days. (A) subjective nighttime activity, (B) number of sleep episodes, and (C) subjective nighttime sleep duration in the DAM. Data are presented as the mean ± the standard error of the mean (SEM) for each group. Different letters indicate significant differences at *p* < 0.05 by Tukey’s mul-tiple comparison test. NOR, normal group; BDZ, positive control group.; Figure S3. Effects of *Dendropanax morbiferus* extract (DE) on mRNA expression associated with antioxidant activity of Drosophila melanogaster. (A) SOD, (B) GPX, and (C) CAT. Values are the means ± standard error of the mean (SEM) for each group. NOR, normal group; BDZ, positive control group; SOD, superoxide dismutase; GPX, glutathione peroxidase; CAT, catalase.
