# Supplementary material for: Effects of Dendropanax morbiferus Leaf Extract on Sleep Parameters in Invertebrate and Vertebrate Models
Source: Antioxidants (Basel). 2023 Oct 21;12(10):1890. doi: 10.3390/antiox12101890 (PMC10604594; doi:10.3390/antiox12101890)
Supplement: Supplementary file 1 [file antioxidants-12-01890-s001.zip › antioxidants-2640140-supplementary.pdf]

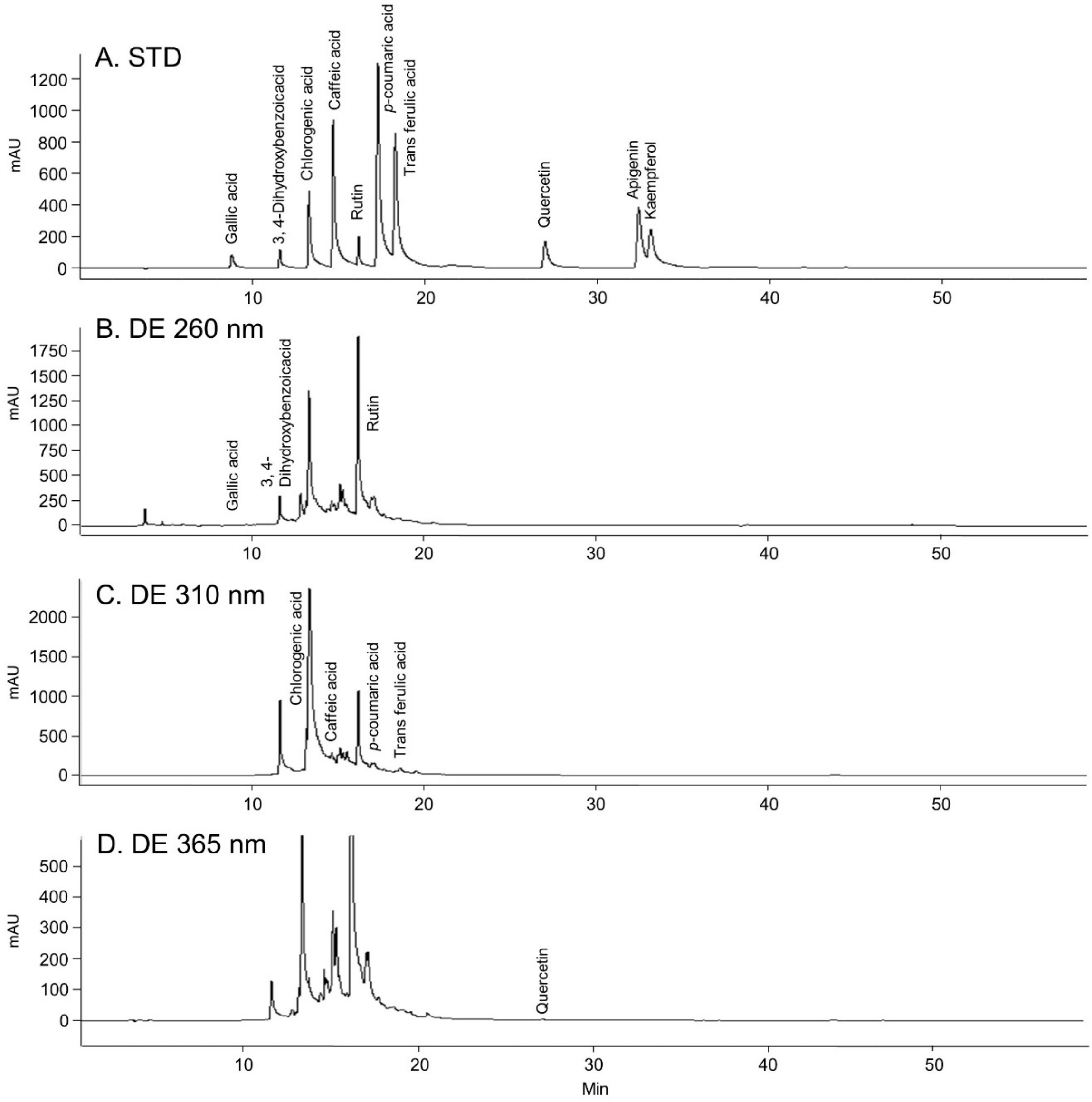

Figure S1. Chromatograms of polyphenols standard and sample using HPLC analysis. A: polyphenols standard, B: gallic acid, 3,4-dihydroxybenzoic acid, and rutin were measured at 260 nm, C: chlorogenic acid, p-coumaric acid, caffeic acid, trans-ferulic acid, and apigenin were measured at 310 nm, D: quercetin and kaempferol were measured at 365 nm.

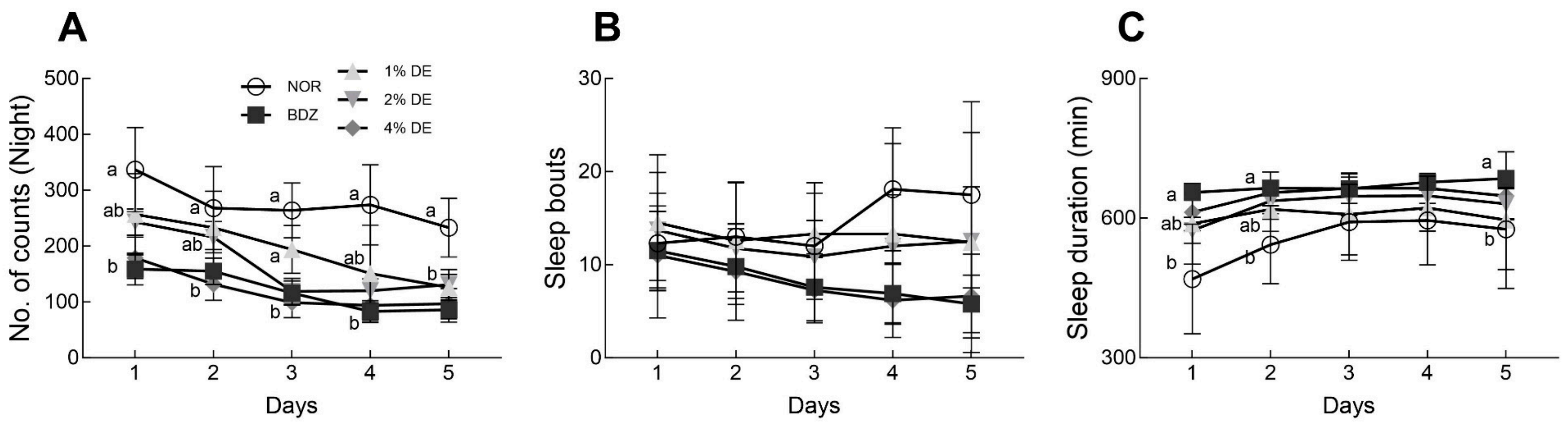

Figure S2. Effects of *Dendropanax morbiferus* extract (DE) on the trend change of sleep activity in *Drosophila melanogaster* over 5 consecutive days. Behavioral analysis was performed after an adaptation period under the light for 1 day and then turning off the light for 5 days. (A) subjective nighttime activity, (B) number of sleep episodes, and (C) subjective nighttime sleep duration in the DAM. Data are presented as the mean ± the standard error of the mean (SEM) for each group. Different letters indicate significant differences at $p<0.05$ by Tukey's mul-tiple comparison test. NOR, normal group; BDZ, positive control group.

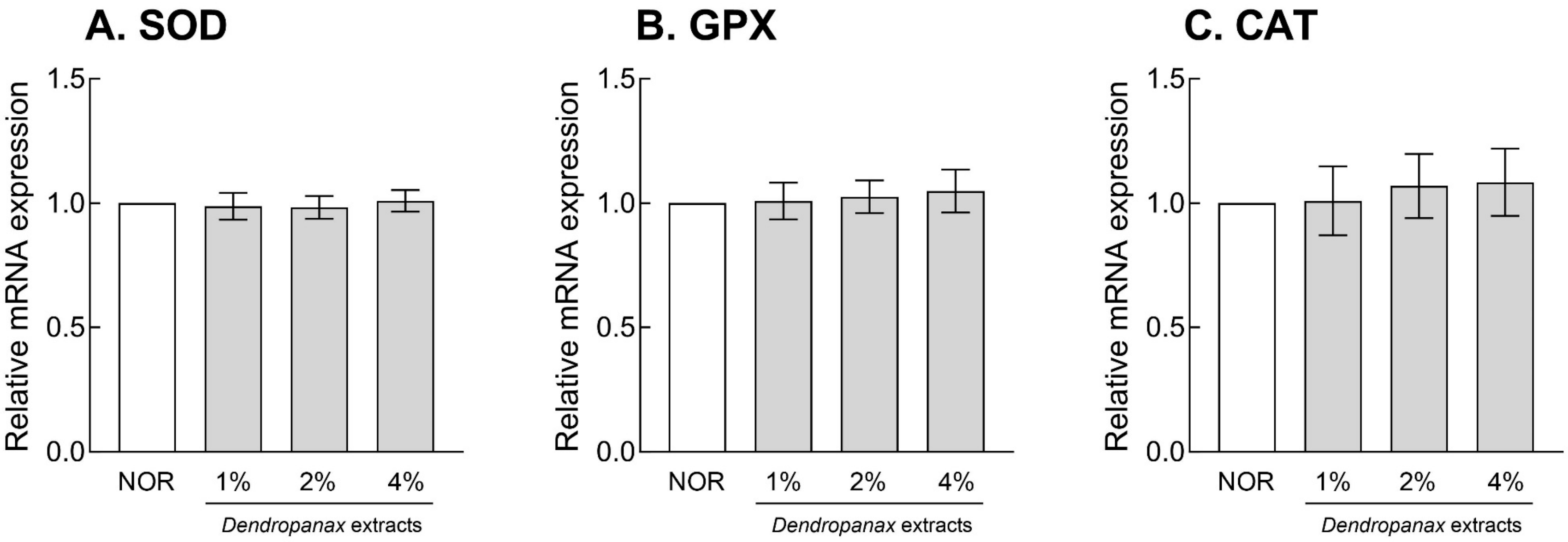

Figure S3. Effects of *Dendropanax morbiferus* extract (DE) on mRNA expression associated with antioxidant activity of *Drosophila melanogaster*. (A) SOD, (B) GPX, and (C) CAT. Values are the means ± standard error of the mean (SEM) for each group. NOR, normal group; BDZ, positive control group; SOD, su-peroxide dismutase; GPX, glutathione peroxidase; CAT, catalase.
